# Development of pH-responsive Eudragit S100-functionalized silk fibroin nanoparticles as a prospective drug delivery system

**DOI:** 10.1371/journal.pone.0303177

**Published:** 2024-05-23

**Authors:** Duy Toan Pham, Doan Xuan Tien Nguyen, Ngoc Yen Nguyen, Thi Truc Linh Nguyen, Thanh Q. C. Nguyen, Anh Vo Thi Tu, Ngoc Huyen Nguyen, Bui Thi Phuong Thuy

**Affiliations:** 1 Department of Chemistry, College of Natural Sciences, Can Tho University, Can Tho, Vietnam; 2 Department of Biology, College of Natural Sciences, Can Tho University, Can Tho, Vietnam; 3 Department of Biostatistics and Demography, Faculty of Public Health, Can Tho University of Medicine and Pharmacy, Can Tho, Vietnam; 4 Faculty of Fundamental Sciences, Van Lang University, Ho Chi Minh City, Vietnam; University of Eastern Piedmont: Universita degli Studi del Piemonte Orientale Amedeo Avogadro, ITALY

## Abstract

Silk fibroin nanoparticles (FNP) have been increasingly investigated in biomedical fields due to their biocompatibility and biodegradability properties. To widen the FNP versatility and applications, and to control the drug release from the FNP, this study developed the Eudragit S100-functionalized FNP (ES100-FNP) as a pH-responsive drug delivery system, by two distinct methods of co-condensation and adsorption, employing the zwitterionic furosemide as a model drug. The particles were characterized by sizes and zeta potentials (DLS method), morphology (electron microscopy), drug entrapment efficiency and release profiles (UV-Vis spectroscopy), and chemical structures (FT-IR, XRD, and DSC). The ES100-FNP possessed nano-sizes of ∼200–350 nm, zeta potentials of ∼ -20 mV, silk-II structures, enhanced thermo-stability, non-cytotoxic to the erythrocytes, and drug entrapment efficiencies of 30%-60%, dependent on the formulation processes. Interestingly, the co-condensation method yielded the smooth spherical particles, whereas the adsorption method resulted in durian-shaped ones due to furosemide re-crystallization. The ES100-FNP adsorbed furosemide via physical adsorption, followed Langmuir model and pseudo-second-order kinetics. In the simulated oral condition, the particles could protect the drug in the stomach (pH 1.2), and gradually released the drug in the intestine (pH 6.8). Remarkably, in different pH conditions of 6.8, 9.5, and 12, the ES100-FNP could control the furosemide release rates depending on the formulation methods. The ES100-FNP made by the co-condensation method was mainly controlled by the swelling and corrosion process of ES100, and followed the Korsmeyer-Peppas non-Fickian transport mechanism. Whereas, the ES100-FNP made by the adsorption method showed constant release rates, followed the zero-order kinetics, due to the gradual furosemide dissolution in the media. Conclusively, the ES100-FNP demonstrated high versatility as a pH-responsive drug delivery system for biomedical applications.

## Introduction

Nanoparticles (NP) have long been investigated as potential drug delivery systems due to their versatility in altering the encapsulated drug pharmacological properties and the ability to be structural modified for various purposes [1]. In most cases, the fabrications of pharmaceutical NP require a carrier, commonly belongs to different classes of inorganic (i.e., Fe_3_O_4_ [2]), lipid (i.e., liposomes [3]), or polymer (i.e., chitosan, poly(lactic-co-glycolic acid) (PLGA) NP [4]). Nevertheless, the inorganic NP might not be human biocompatible [5] and the lipid-based NP are generally unstable [6]. These obstacles, together with the recent "back-to-nature" trend, naturally-derived substances/materials such as silk fibroin are attractive carriers for NP formulations, having enhanced biocompatibility, biodegradability with non-toxic products, cost-effectiveness, and ease of manufacturing [7–9].

Fibroin, an FDA-approved biomaterial generally obtained from the silk fibers of the domestic *Bombyx mori* silkworm through a sericin-removal degumming process, possesses excellent properties of human biocompatibility, mucoadhesiveness, non-toxicity, and preparation flexibility [7, 10–13]. Therefore, fibroin has been used in a variety of drug delivery systems, including hydrogels, films, microparticles, and NP, for administrations via numerous routes of oral, parenteral, transdermal, and ophthalmic [8, 14–19]. Interestingly, fibroin maintains its high stability across a broad pH range from less than 5 to 14 [7], making FNP suitable for utilizations in harsh conditions such as the gastrointestinal tract [20], the cancer microenvironment [21], and the inflammatory areas [22]. However, the FNP ability to precisely control the encapsulated drug release rates in different pH is limited, possibly due to the weak drug-material interactions and the loosely packed structure of fibroin in FNP [23, 24]. To overcome this disadvantage, one potential approach is to functionalize the FNP with a pH-responsive polymer such as Eudragit S100 (ES100).

The utilizations of polymers for FNP functionalization, namely poly(ethylene glycol) (PEG), poly(vinyl alcohol) (PVA), and poly(ethylenimine) (PEI), have resulted in remarkable advantages, including controllable particle sizes, enhanced drug loading efficiencies, fortified particle structures, sustained drug releases, and safeguarded drug degradations [25–28]. During the functionalization processes (i.e., coating, incorporating), additional interactions between fibroin and polymers are created, including (1) the van der Waals forces between large fibroin and polymer molecules, (2) the ionic interactions between the negatively charged fibroin and the positively charged polymers, (3) the hydrogen bonding between the hydrogen atoms and oxygen/nitrogen atoms in the fibroin and polymers, and (4) the hydrophobic interactions between the repetitive Gly-Ser-Gly-Ala-Gly-Ala amino acid sequences of fibroin and the lipophilic sections of polymers). These interactions significantly and favorably alter the newly formed polymer-FNP complex properties, consequently enhancing the particles behaviors. Nevertheless, to the best of our knowledge, no in-depth investigation has been reported on the FNP functionalization with a pH-response polymer such as ES100.

ES100, an anionic copolymer (molecular weight of ∼135,000 Da) composed of methacrylic acid and methyl methacrylate with the ratio of the free carboxyl groups to the ester groups of 1:2, has its solubility heavily depended on the solution pH (i.e., low solubility at pH ≤ 7, high solubility at pH > 7). Due to its unique property, ES100 is commonly used as a tablet coating agent for the purposes of colon-specific drug delivery [29, 30] and sustained-release systems [31]. Recently, ES100 has been exploited as pH-responsive micro-/nanoparticulate systems for biomedical applications, either by itself [32, 33] or in combination with other polymers such as PLGA [34], dextran [35], and chitosan [36]. Nevertheless, these reports did not mention the versatility of ES100 in encapsulating, adsorbing, and releasing the drugs at different pH. Moreover, ES100 has not been functionalized with FNP.

Therefore, the study main objective was to develop the ES100 functionalized FNP (ES100-FNP) and FNP (the non-functionalized counterpart), encapsulating the drug furosemide, as a novel control-released pH-responsive drug delivery system. Furosemide was selected due to its zwitterionic property, with two pKa values of ∼3.8 and ∼10 [37], thus, its charges alter in different pH and could interact differently with the ES100-FNP, consequently resulting in the distinctive particles properties such as the release rates. Moreover, furosemide is classified as a biopharmaceutical classification system (BCS) class-IV (low solubility and low permeability characteristics), with an inadequate oral bioavailability [38]. Hence, these class-IV drugs commonly need to be administrated with the help of a drug delivery system (i.e., NP) to improve their pharmacokinetics properties. The particles were fabricated by adsorption and co-condensation method, underwent physicochemical characterizations of particle sizes, polydispersity, zeta potentials, shape/morphology, drug entrapment efficiency, particle structures, and hematotoxicity. Next, the mechanisms underlying the drug adsorption and release behaviors in different pH conditions were investigated by different analytical techniques and suitable mathematics models.

## Materials and methods

### Materials

*Bombyx mori* cocoons, variety M45, were procured from Nam Dinh, Vietnam. Sheep blood, which served as a source of red blood cells, was obtained from a local slaughterhouse in Can Tho, Vietnam, stored in specialized bags containing sodium citrate, and was utilized within a week of procurement. Furosemide was bought from Trung Son pharmacy, Can Tho, Vietnam. ES100 was imported from Evonik, Germany. The molecular structures of silk fibroin, ES100, and furosemide are demonstrated in **S1 Fig**. Other general chemicals/solvents were of reagent grades or higher.

### Silk fibroin extraction

The process of extracting and refining regenerated fibroin from silk cocoons was conducted with reference to a prior publication [39]. In brief, the silk cocoons (10 g) underwent sericin-removal process with 200 mL Na_2_CO_3_ (0.5% w/v) at 100°C for 1 h, washed thrice with water, and air-dried. The resulting sericinized silk (5 g) was then dissolved in a mixture (50 mL) of CaCl_2_:H_2_O:Ca(NO_3_)_2_:EtOH (30:40:5:20 w/w/w/w) at 90°C for 5 min to obtain the fibroin solution. The solution was dialyzed against water at room temperature for 3–5 days, removed insoluble solids by centrifugation (18000 rpm, 4°C, 30 min) (Mikro 220R, Hettich, Germany), and freeze-dried. The final obtained water-soluble regenerated fibroin powder (silk-I polymorph) was stored at 4°C for further uses.

### Formulations of the blank FNP and ES100-FNP

The blank FNP and ES100-FNP (i.e., particles without drugs) were formulated by the condensation method [28]. For this, 1 mL of pure ethanol was added to 1 mL of the fibroin solution (1% w/v), with or without the addition of 1 mL ES100 ethanolic solution (1% w/v). The mixture was stabilized at 4°C for 1 h, and the formed NP was collected by centrifugation (18000 rpm, 30 min), washed thrice with water, and freeze-dried for further experiments.

### Formulations of furosemide loaded FNP and ES100-FNP

The furosemide loaded FNP (FNP-Fu) and ES100-FNP (ES100-FNP-Fu) were formulated by two methods, including the co-condensation method and the adsorption method. For the co-condensation method, the particles were fabricated similarly to the preparation of the unloaded particles, with the replacement of “1 mL of pure ethanol” with “1 mL of pure ethanol containing 1 mg or 5 mg furosemide”.

For the adsorption method, 0.015 g of the blank FNP or blank ES100-FNP was dispersed in 20 mL of furosemide solution (250 μg/mL), followed by continuous stirring for 4 h. The generated drug-adsorbed NP was collected by centrifugation (18000 rpm, 30 min), washed thrice with water, and freeze-dried for further experiments.

Nanoparticle characterizations

#### Particle size, polydispersity, and zeta potential

The average size of particles and their distribution, known as the polydispersity index (PI), were assessed utilizing the dynamic light scattering (DLS) technique with the employment of the ZetaSizer^®^ analyzer (SZ-100 Horiba, Japan). PI is a unitless number derived by dividing the square of the standard deviation by the square of the average particle sizes, calculated by the machine included software. A higher PI suggests greater variance in particle size within the system, in which a PI below 0.1 signifies a uniform distribution, while a value below 0.5 indicates a relatively narrow distribution, and a PI exceeding 0.5 suggests a wide distribution [40]. The zeta potential was determined using the same instrument, employing the phase analysis light scattering (PALS) method. For both measurements, the samples were diluted with water until a count rate of 400–600 kcps and the machine operations were followed the instruction manuals at room temperature.

#### Morphology

The morphology of the particles was observed using scanning electron microscope (SEM, Carl Zeiss, Germany). The particles were dispersed in water and diluted until reaching a count rate of 400 kcps, as determined through the DLS method. Subsequently, the dispersions were deposited and immobilized onto SEM stubs, coated with a 10-nm thick gold layer, and subjected to SEM analysis.

#### Drug entrapment efficiency

To determine the drug entrapment efficiency (EE%) of the FNP-Fu and ES100-FNP-Fu, after the formulation processes, the particles were centrifuged and the unencapsulated furosemide in the supernatant was measured by UV-Vis spectroscopy at a wavelength of 234.5 nm (Jasco V730, Japan), and calculated by a calibration curve (y = 0.1334x + 0.0497, R^2^ = 0.9971, range 0–10 μg/mL). The EE% was determined using Eq (1).


$$\text{EE}\%=\frac{\text{The}\;\text{initial}\;\text{furosemide}\;\text{amount}-\text{The}\;\text{unencapsulated}\;\text{furosemide}\;\text{amount}}{\text{The}\;\text{initial}\;\text{furosemide}\;\text{amount}}\times 100\;(\%)$$ (1)

#### Drug adsorption process

To follow the furosemide adsorption process onto the FNP and ES100-FNP, 1 mL of the adsorption mixtures was withdrawn at 30-min intervals for the entire 4-h process, medium refilled, centrifuged (18000 rpm, 5 min), and the unadsorbed furosemide in the supernatant was analyzed using UV-Vis spectroscopy, as described in **section Drug entrapment efficiency**. The percentages of furosemide adsorption, at each time point, was calculated by Eq (2)

$$\%\text{Adsorption}=\frac{\text{Initial}\;\text{furosemide}\;\text{concentration}\;(250\mu \text{g}/\text{mL})-\text{Unadsorbed}\;\text{furosemide}\;\text{concentration}}{\text{Initial}\;\text{furosemide}\;\text{concentration}\;(250\mu \text{g}/\text{mL})}\times 100\;(\%)$$ (2)

Additionally, the furosemide adsorption isotherms and kinetics were evaluated using the Langmuir model (Eq (3)) and Dubinin-Radushkevich model (Eq (4)), and the pseudo-first-order model (Eq (5)) and the pseudo-second-order model (Eq (6)), respectively.

$$\frac{q_{e}}{q_{m}}=\frac{K_{L}C_{e}}{1+K_{L}C_{e}}$$ (3)

$$\text{ln}q_{e}=\ln q_{m}-\beta \varepsilon^{2}$$ (4)

$$\text{ln}\left({\text{q}}_{\text{e}}-{\text{q}}_{\text{t}}\right)=\ln\left({\text{q}}_{\text{e}}\right)-{\text{k}}_{1}\text{t}$$ (5)

$$\frac{t}{q_{t}}=\frac{1}{k_{2}q_{e}^{2}}+\frac{t}{q_{e}}$$ (6)

where C_e_ is the furosemide equilibrium concentration (mg/L), q_e_, q_m,_ q_t_ are the adsorption capacities at equilibrium, at maximum values, and at time point t (mg/g), respectively, K_L_, β, k_1_, k_2_ are the Langmuir constant (L/mg), the Dubinin-Radushkevich constant (mol^2^/kJ^2^), and the apparent first-order and second-order adsorption rate constant, respectively, and $\varepsilon =\text{RT}\ln\;\left(1+\frac{1}{\text{Ce}}\right)$ is the Polanyi potential energy.

#### Particles pHpzc analysis

Since fibroin molecules possess both positive charges at free amines groups and negative charges at free carboxylic groups, the pHpzc (pH at point of zero charge) is a crucial factor to explain the particles-furosemide adsorption mechanisms. For this reason, the pHzpc of FNP and ES100-FNP was conducted by the standard solid addition technique [41]. Ten mg of the freeze-dried particles were re-dispersed in 20 mL KCl 0.1 M at different initial pH (pH_i_) of 1, 2, 4, 6, 8, 10, and 12 (pH adjustments by HCl 0.1 M or NaOH 0.1 M). The mixtures were then stirred (150 rpm, 25°C, 24 h) and the solutions final pH (pH_f_) were re-measured. Finally, the graphs were plotted between the ΔpH (pH_f_−pH_i_) against the initial pH, and the point at which ΔpH = 0 was the pHpzc of the NP. For comparison purpose, the pHpzc of the pure furosemide (pKa) was also determined.

#### Particle structure

The structures and drug-material interactions of the blank FNP, blank ES100-FNP, FNP-Fu, and ES100-FNP-Fu, were determined using the Fourier-transform infrared spectroscopy (FT-IR), X-ray diffractometry (XRD), and differential scanning calorimetry (DSC) analyses. The FT-IR spectra were recorded by a Jasco 6300 spectrophotometer (Japan), using the standard KBr pelleting method, with a wavenumber range of 4000–400 cm^-1^, at a resolution of 4.0 cm^-1^.

The XRD graphs were measured on a D8 Advance XRD instrument (Bruker, USA), using Cu Kα radiation (45 kV, 36 mA, λ = 0.154 nm). The particles were spread out onto quartz substrates and analyzed from 5 to 50° (2θ) at a scan speed of 2°/min.

The DSC analysis was performed on the DSC 200 F3 machine (Netzsch, Germany) equipped with a ceramic-FRS6 sensor, in a temperature range of 40–240°C, with a scanning rate of 3 K/min, under a nitrogen gas flow of 20 mL/min. The particles (5 mg) were subjected to an aluminum pan and measured with a blank pan as a reference.

#### Hematotoxicity

The hematotoxicity of the blank FNP, blank ES100-FNP, FNP-Fu, and ES100-FNP-Fu was *in vitro* examined by determining the hemolysis percentage on sheep red blood cells [21, 42]. Briefly, the sheep whole blood was centrifuged (4000 rpm, 5 min), and the accumulated erythrocytes were re-dispersed and washed thrice with phosphate buffer saline (PBS) and diluted with PBS to a concentration of 1% w/v (1% hematocrit). Following this, the particles, at various concentrations of 1, 2.5, 5, and 10 mg/mL, were incubated with 1 mL of the erythrocytes at 37°C for 30 min. Finally, the samples were centrifuged (4000 rpm, 5 min), the hemoglobin in the supernatants was UV-Vis spectroscopic measured at 540 nm, and the hemolysis (%) was determined by Eq (7).

$$\%\text{Hemolysis}=\frac{A_{\text{nanoparticles}}-A_{PBS}}{A_{\text{water}}-A_{PBS}}\times 100\;(\%)$$ (7)

where A_nanoparticles_, A_water_, and A_PBS_ are the absorbance values of the erythrocytes treated with NP test samples, with water (the positive control), and with PBS (the negative control).

### *In vitro* drug release study

To investigate the ability to release furosemide of FNP-Fu and ES100-FNP-Fu, the *in vitro* release tests were carried out using the standard shaking method [28] at 37°C in different pH conditions, including the gastrointestinal tract (HCl pH 1.2 for 2 h, followed by PBS pH 6.8 for 6 h), pH 6.8, pH 9.5, and pH 12.

For the release test in the simulated gastrointestinal tract condition, the FNP-Fu and ES100-FNP-Fu (0.01 g) were dispersed in 10 mL HCl pH 1.2, simulating the stomach fluid, and shaken for 2 h. Subsequently, the NP were transferred to a 40-mL solution of PBS at pH 6.8, simulating the intestinal fluid, for an additional 6 h. At intervals of 30 min, 1 mL of the mixture was aspirated and medium replaced. The aspirates were centrifuged (18000 rpm, 3 min) and the supernatants were UV-Vis spectroscopic measured.

For the release tests in other pH (i.e., pH 6.8, pH 9.5, pH 12), the FNP-Fu and ES100-FNP-Fu (0.01 g) were dispersed in 40 mL of phosphate buffers with respective pH and continuously shaken for 8 h. At intervals of 30 min, 1 mL of the mixture was aspirated and medium replaced. The aspirates were centrifuged (18000 rpm, 3 min) and the supernatants were UV-Vis spectroscopic measured.

The furosemide maximum absorption wavelengths (λ_max_) and calibration curves in different media are represented in **Table 1**. The cumulative furosemide release was calculated using Eq (8).

$$\%\text{Furosemide}\;\text{release}=\frac{C_{t}V_{0}+v\sum_{1}^{t-1}c_{i}}{M_{0}-\sum_{1}^{t-1}M_{i}}\times 100\;(\%)$$ (8)

where C_t_, C_i_ are the released furosemide concentrations at the time point t and i, V_0_ is the medium total volume, V is the withdrawal volume (1 mL), and M_0_, M_i_ are the initial furosemide amount and withdrawal furosemide amount at the time point i.

**Table 1:** Maximum absorption wavelengths (λ_max_) and calibration curves of furosemide release in simulated media at different pH.

Medium pH	λ_max_ (nm)	Calibration curve
1.2	233.5	y = 0.1343x - 0.0029
		R² = 0.9988, range 1–10 μg/mL
6.8	229.5	y = 0.1015x + 0.0114
		R² = 0.9989, range 1–10 μg/mL
9.5	229	y = 0.1004x + 0.0066
		R² = 0.9999, range 1–10 μg/mL
12	228	y = 0.1033x + 0.0027
		R² = 0.9995, range 1–10 μg/mL

To evaluate the drug release kinetics, general mathematical models were employed, including the zero-order (Eq (9)), first-order (Eq (10)), Higuchi (Eq (11)), Korsmeyer-Peppas (Eq (12)), and Hixson-Crowell (Eq (13)).

$${\text{M}}_{\text{t}}={\text{M}}_{0}+{\text{k}}_{0}\text{t}$$ (9)

$$\ln\left({\text{M}}_{\text{t}}\right)=\ln\left({\text{M}}_{0}\right)-{\text{k}}_{1}\text{t}$$ (10)

$$M_{t}=k_{H}t^{1/2}$$ (11)

$$\frac{{\text{M}}_{\text{t}}}{{\text{M}}_{0}}={\text{k}}_{\text{KP}}{\text{t}}^{\text{n}}$$ (12)

$${\text{M}}_{\text{t}}^{1/3}={\text{M}}_{0}^{1/3}-{\text{k}}_{\text{HC}}\text{t}$$ (13)

where M_0_, M_t_ are the initial amount of furosemide and the released furosemide amount at the time point t, k_0_, k_1_, k_H_, k_KP_, and k_HC_ are the release constants of the zero-order, first-order, Higuchi, Korsmeyer-Peppas, and Hixson-Crowell (constant incorporating the surface-volume relation) models, respectively.

The zero-order model indicates that the rate of drug release is independent of the drug concentration in the NP (i.e., constant amount of drug is released in a time-dependent manner), whereas the first-order kinetics describes a release rate proportional to the amount of drug remaining in the NP (i.e., the release rate decreases as the drug concentration decreases over time). On the other hand, the Higuchi model, based on Fick’s law of diffusion, expresses the drug release amount as a square root of time-dependent process, which suggests that the rate of drug release is directly proportional to the concentration gradient within the NP matrix. The Korsmeyer-Peppas model characterizes non-Fickian release, mostly from polymeric systems, including those exhibiting swelling or erosion properties, with imply a complex release profile of an initial burst release followed by a sustained release. Finally, the Hixson-Crowell model describes drug release based on the dissolution or degradation of the NP matrix with surface area/geometry alteration over time [43].

### Nanoparticle characterizations after drug release study

After the release tests in different pH of 6.8, 9.5, and 12, the remaining FNP-Fu and ES100-FNP-Fu in the media were determined their properties, focused on the particle structures. To this end, the NP were collected by centrifugation (18000 rpm, 5 min), freeze-dried, and subjected to FT-IR analyses as described in **section Particle structure**. The NP were evaluated two parameters, the crystallinity and the FT-IR characterized peak intensities.

The NP crystallinity was determined based on the crystallinity index (CI, the ratio of the NP crystalline portion and the sum of NP amorphous and crystalline portions). The FNP and ES100-FNP CI were calculated according to the FT-IR signal intensities of the fibroin characterized amide I peak, by Eq (14) [24].

$$CI=I_{1622}/(I_{1622}+I_{1646})$$ (14)

where I_1622_ and I_1646_ are the signal intensities of the fibroin crystalline portions and amorphous portions at amides I peak.

Additionally, the ES100 characterized peak at ∼1045 cm^-1^ (C-O stretching) and furosemide characterized peak at ∼880 cm^-1^ (aryl-chloro group oscillation) were also compared between the initial ES100-FNP-Fu and the particles after the release studies.

### Statistical analysis

The quantitative studies were carried out in triplicate, the respective data were reported as mean ± standard deviation (SD), and Student’s *t*-test and one-way analysis of variance were conducted for statistical comparisons, with a set *p* value of < 0.05.

## Results and discussions

### Particle size, polydispersity, and zeta potential

Prior to the formulations of FNP-Fu and ES100-FNP-Fu, the blank counterparts were fabricated. For this, both the blank FNP and blank ES100-FNP were successfully synthesized with particle sizes in the nanometer range with narrow size distributions (PI < 0.3) (**Table 2**). Interestingly, the addition of ES100 to the fibroin systems led to the generation of NP with comparatively smaller sizes (∼320 nm for FNP and ∼220 nm for ES100-FNP), in agreement with the previous study of Seremeta *et al*., which also observed this trend in poly(ε-caprolactone)/Eudragit blend NP [44]. This was because ES100 could induce steric hindrance effect that stabilizes the FNP and slows down the fibroin aggregation process, thus creating smaller-sized particles. Moreover, the zeta potential of ES100-FNP was higher (i.e., more negative) than that of the FNP, which was due to the additive negative charges of the ES100 carboxylic groups at pH 7.0.

**Table 2:** Particle sizes, polydispersity indexes (PI), and zeta potentials of the blank FNP, blank ES100-FNP, FNP-Fu, and ES100-FNP-Fu, at two encapsulation methods of co-condensation and adsorption (n = 3).

Formulation	Particle size (nm)	PI	Zeta (mV)
**Blank particles**			
FNP	327.14 ± 19.32^a^	0.212 ± 0.059^a^	-16.76 ± 0.53^a^
ES100-FNP	218.60 ± 20.28^b^	0.208 ± 0.073^a^	-25.90 ± 0.42^b^
**Furosemide loaded particles**			
**Co-condensation**			
FNP-Fu	480.81 ± 11.36^c^	0.193 ± 0.069^a^	-10.92 ± 1.34^c^
ES100-FNP-Fu	250.78 ± 16.15^b,d^	0.224 ± 0.042^a^	-18.44 ± 1.15^d^
**Adsorption**			
FNP-Fu	532.63 ± 18.65^e^	0.201 ± 0.080^a^	-11.37 ± 0.96^c,e^
ES100-FNP-Fu	339.18 ± 14.96^a,f^	0.199 ± 0.038^a^	-18.03 ± 0.78^d,f^

^a-f^Different letters denote significant differences between values in the same column (p < 0.05).

Then, the model drug furosemide was incorporated into the FNP and ES100-FNP by two different methods of co-condensation and adsorption. For both methods, the particles sizes significantly increased compared to the blank counterparts, suggesting the success drug encapsulations [21] (**Table 2**). Moreover, particles fabricated by the adsorption method were larger than those made by the co-condensation, which was due to the partly re-crystallization of the furosemide on the particle surfaces, discussed in the next sections.

### Morphology

**Fig 1:**
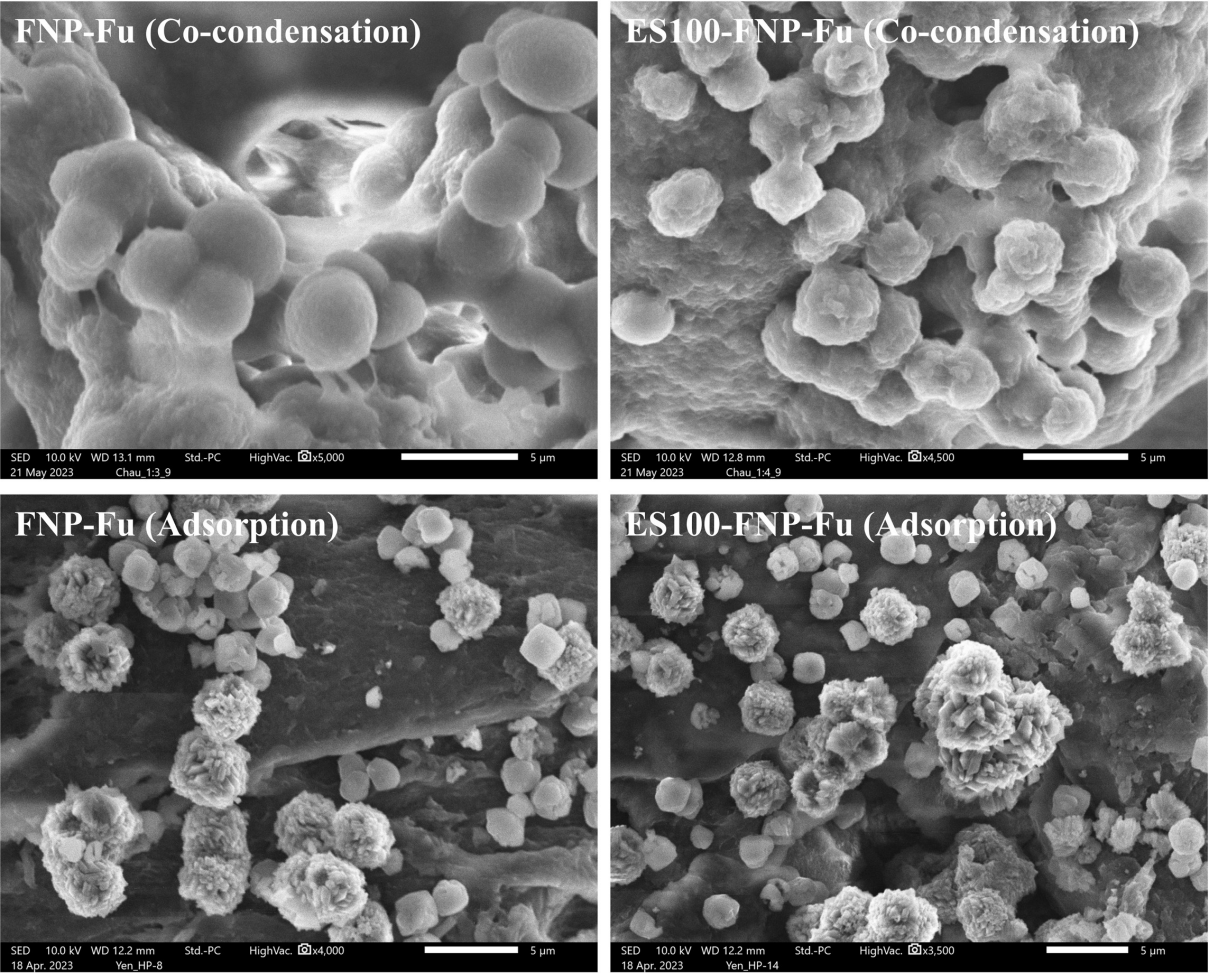
Scanning electron microscopy (SEM) micro-images of FNP-Fu and ES100-FNP-Fu at two encapsulation methods of co-condensation and adsorption.

The SEM micro-images were used to observe the morphology of the FNP-Fu and ES100-FNP-Fu, formulated by both co-condensation and adsorption methods (**Fig 1**). Firstly, it is worth to note that the particles sizes observed by the SEM were different than those measured by the DLS method, which could be due to the variations in sample preparations, analytical procedures, and instrumental issues [45]. Secondly, regarding the particle morphology, for the co-condensation method, the FNP-Fu possessed spherical particles with smooth surfaces, in accordance with previous works [20, 42]. On the other hand, the ES100-FNP-Fu showed rougher surfaces with multilayer coatings, indicating the success ES100 functionalization on the FNP. Interestingly, the NP made by the adsorption process demonstrated durian-shaped structures, of which the “durian thorns” are the adsorbed furosemide that partly re-crystallized on the particle surfaces. To the best of our knowledge, this is the first time the durian-shaped NP was discovered in the drug delivery area. These “thorns” were beneficial for the controlled drug release in different pH, discussed in **section *In vitro* drug release study**.

The differences between these formulas come from different formulation processes. In the co-condensation method, the furosemide molecules were in the solution form (i.e., dissolved in ethanol), and only the fibroin precipitated (i.e., silk-I to silk-II structural transformation) during the condensation process due to the solvent adjustment from water to ethanol [7]. As a consequence, the furosemide molecules were homogenously dispersed, in the form of molecular dispersion, all over the particles (i.e., both inside and on the surfaces of the particles), resulting in smooth NP. Conversely, in the adsorption method, the blank particles were immersed in the furosemide solution for 4 h. Thus, the drug molecules were gradually adsorbed onto the NP surfaces, and these adsorbed molecules interacted with each other [37], forming aggregated clusters of furosemide, ultimately re-crystallized the drugs. Therefore, the “durian thorns” were generated.

### Drug entrapment efficiency

**Fig 2:**
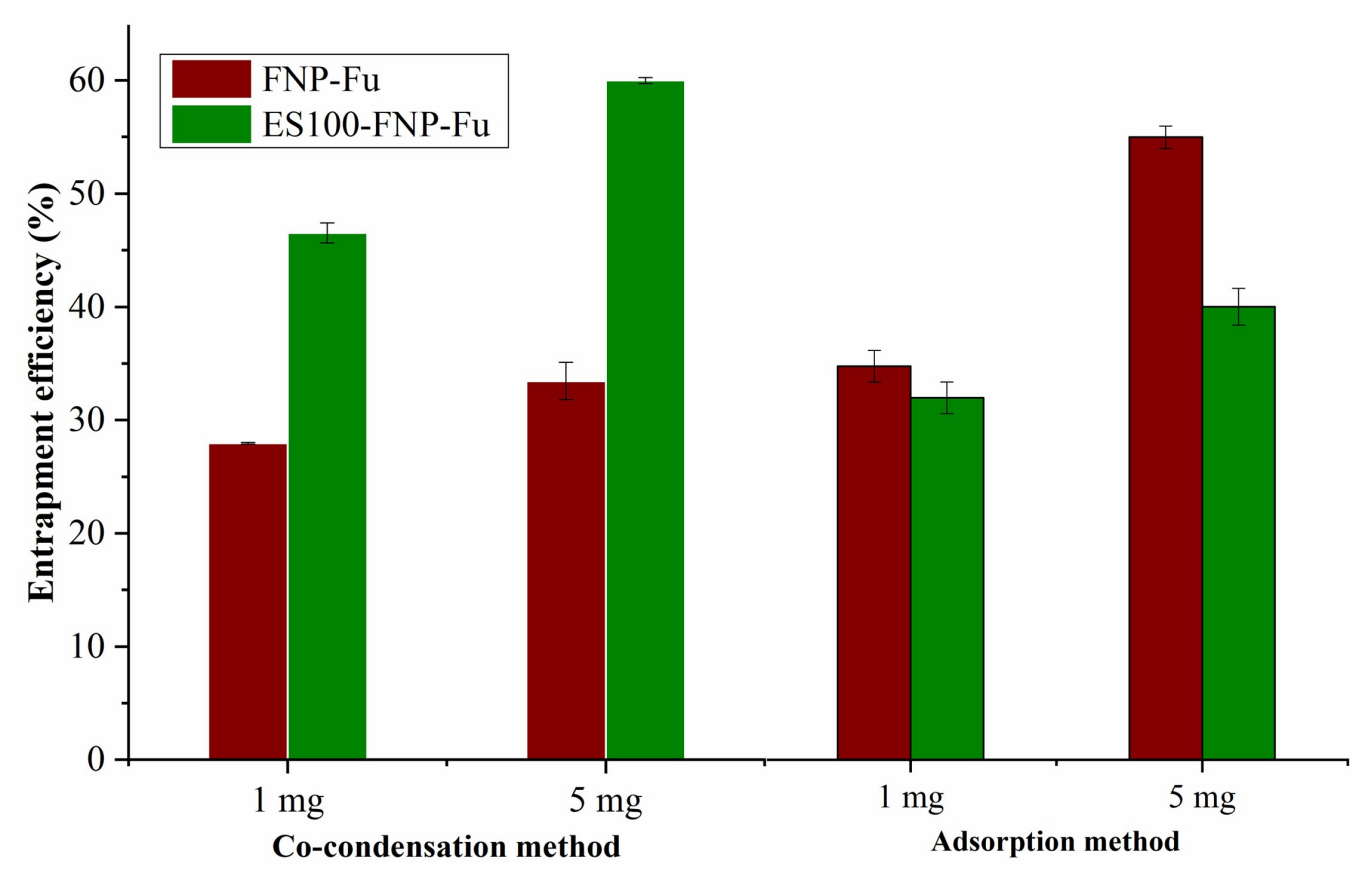
The entrapment efficiency (%) of FNP-Fu and ES100-FNP-Fu with a furosemide loading content of 1 mg and 5 mg, by two encapsulation methods of co-condensation and adsorption (n = 3).

The furosemide EE% were determined in four formulas, at two initial drug amounts of 1 mg and 5 mg, which ranged from ∼30% to ∼60%, dependent on the formulas (**Fig 2**). Obviously, the EE% at 5-mg initial furosemide were higher than those at 1 mg, at all formulas. This suggests that the FNP and ES100-FNP reached their saturation point (i.e., the maximum limit of the particles/materials on encapsulating drugs) at the initial dose of 5 mg [21, 42]. Thus, to get the highest possible encapsulated drug amounts, the 5-mg initial furosemide amount was selected.

Interestingly, when compared between the FNP and ES100-FNP, the EE% of the ES100-FNP-Fu was significantly higher than those of the FNP-Fu in the co-condensation method, yet this issue was reversed in the adsorption method (**Fig 2**). This might be related to interactions between the functional groups of fibroin, ES100, and furosemide. Specifically, the O-H group (carboxylic acid) and hydrogen atoms bonded to carbon atoms in the chain structures of ES100 can form additional hydrogen bonding with the -NH_2_ group of furosemide and the amino acid residues of fibroin. Consequently, more furosemide molecules were entrapped in the ES100-FNP system. However, ES100 could also act as a surfactant and a solubilizer [46], leading to an increase in furosemide aqueous solubility, thereby decreasing the amount of drug encapsulated in the fabricated NP. In summary, the incorporation of ES100 greatly influences the EE% of FNP formulations, which could be critically considered in future studies.

### Drug adsorption process

**Fig 3:**
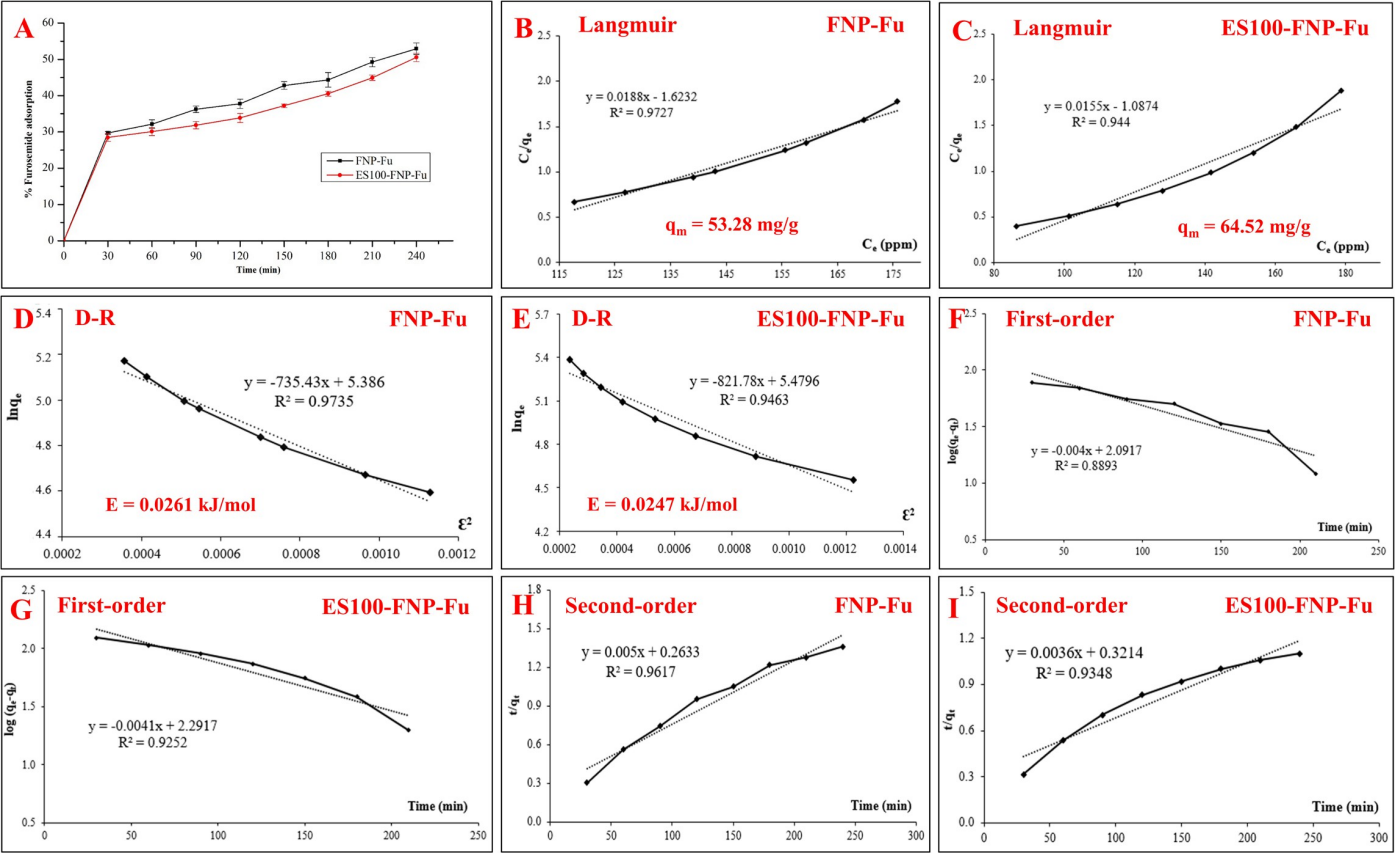
Furosemide adsorption (%) onto FNP-Fu and ES100-FNP-Fu (A) and the underlying adsorption mechanisms, regarding the isotherm Langmuir model (B, C) and Dubinin-Radushkevich (D-R) model (D, E), and kinetics pseudo-first-order model (F, G) and pseudo-second-order model (H, I).

**Fig 4:**
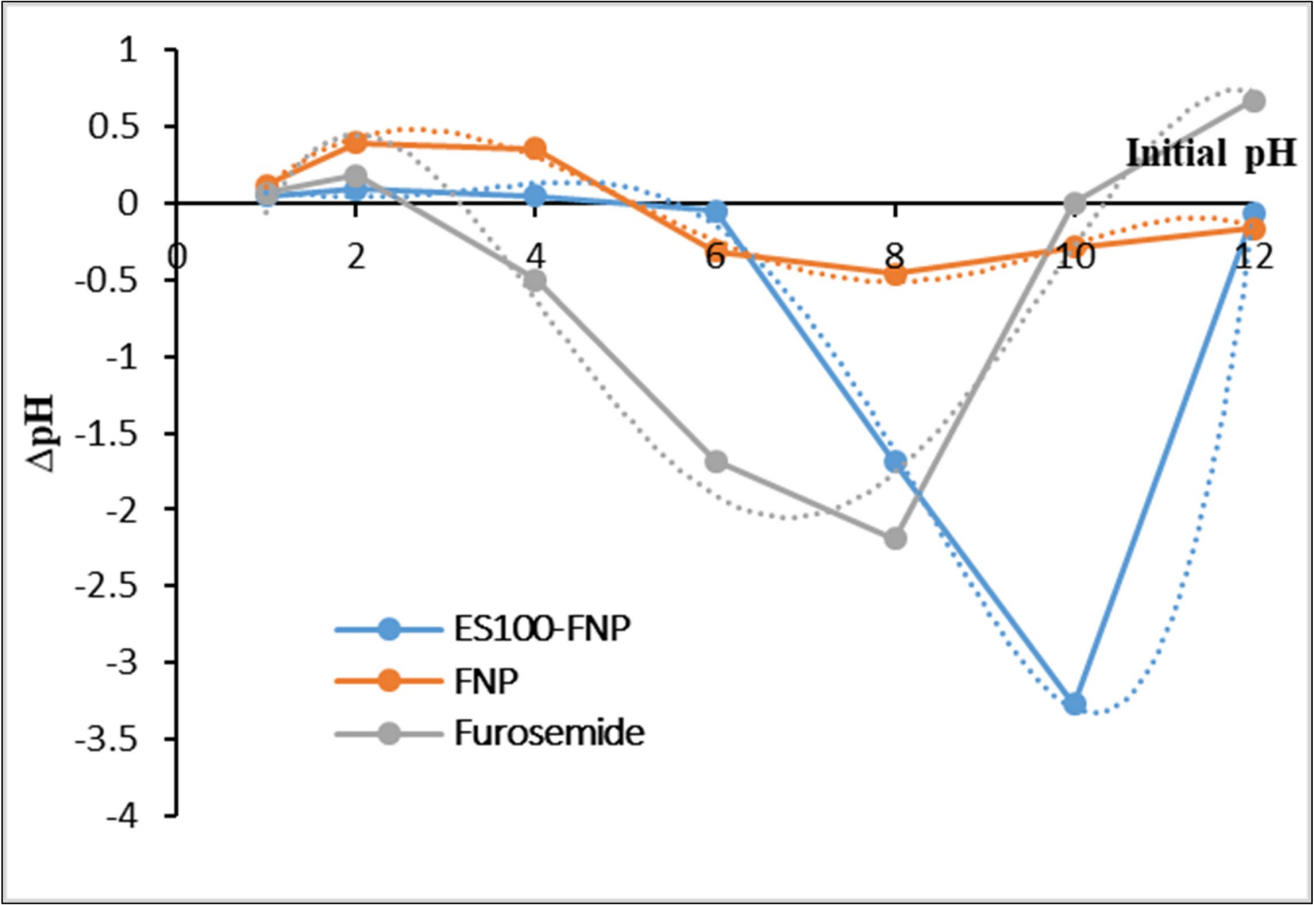
Graphs of pH at point of zero charge (pHpzc) of the FNP, ES100-FNP, and the pure furosemide.

Since the furosemide adsorption process onto the FNP and ES100-FNP surfaces yielded unique structures, in-depth analyses of this process were conducted, including (1) the adsorption profile (**Fig 3A**), (2) the adsorption isotherms and kinetics (**Fig 3B–3I**), and (3) the particles-furosemide adsorption mechanisms, by pHpzc investigation (**Fig 4**).

Firstly, the furosemide adsorptions underwent two distinct phases, with the first phase being rapid adsorption within the first 30 min, followed by the second slow-adsorption phase for the remaining time (**Fig 3A**). This fact further clarifies the previous discussion on the “durian thorns” formation, in which the furosemide molecules were gradually deposited on the NP surfaces, consequently interacting with each other and generating furosemide crystals.

Secondly, isothermal modelling shows that both the FNP-Fu and ES100-FNP-Fu followed the Langmuir isotherm model, with a maximum adsorption capacity of 53.28 mg/g for FNP-Fu and 64.52 mg/g for ES100-FNP-Fu (**Fig 3B and 3C**), indicating that the adsorption process was mainly monolayer [47]. Moreover, these theoretical values were less than those of the actual capacity (∼176.3 mg/g for FNP-Fu and 168.5 mg/g for ES100-FNP-Fu), which were due to the additional adsorption of furosemide molecules onto the other furosemide layers. Furthermore, the D-R isotherm model (**Fig 3D and 3E**), with the average adsorption energy E (calculated as $\text{E}=\frac{1}{\sqrt{2\beta }}$) of 0.0261 kJ/mol for FNP-Fu and 0.0247 kJ/mol for ES100-FNP-Fu, suggested that the adsorption process was primarily occurred via physical interactions [28] with van der Waals forces and hydrogen bonding between the -COOH group of furosemide and the -NH group of the fibroin, as well as the O-H group (carboxylic acid) of ES100. Last but not least, the furosemide adsorption kinetics followed well with the pseudo-second-order model, rather than the pseudo-first-order model (**Fig 3F–3I**). This indicates that the furosemide adsorption rate was mostly dependent on the adsorption capacity of the FNP and ES100-FNP, but not on the furosemide concentrations [48].

Thirdly, the pHpzc graph (**Fig 4**) demonstrates that the pHpzc (i.e., the pH point of which the substance/material possesses zero net charge) of the FNP, ES100-FNP, and furosemide was ∼5.0, ∼5.4, and ∼3.2 and ∼10.2, respectively. The furosemide pHpzc was similar to those reported in the previous study [37]. Thus, at the adsorption pH of 7.0, the particles were mainly in the negative charged state, due to the amine residue groups in the fibroin molecules [7], and the furosemide possessed both the negative (COO-) and positive (NH_3_^+^) charges. Hence, ionic interactions could form between the negatively charged particles and the positively charged moieties of furosemide. Moreover, the furosemide negative and positive charged moieties could accelerate the ionic interactions between the drug molecules themselves, making more drugs stayed on the particle surfaces, consequently induce the re-crystallization process that formed the “durian thorns”.

Conclusively, the furosemide adsorption processes onto the FNP and ES100-FNP (1) were physical adsorption, with van der Waals forces, hydrogen bonding, and ionic interactions; (2) occurred in two phases, the initial rapid adsorption and the slow-adsorption phase, with the main factor governed the adsorption rate was the particle adsorption capacity; and (3) followed the Langmuir monolayer-adsorption isotherm model with fragmented layers of furosemide being re-crystallized.

### Particle structure

**Fig 5:**
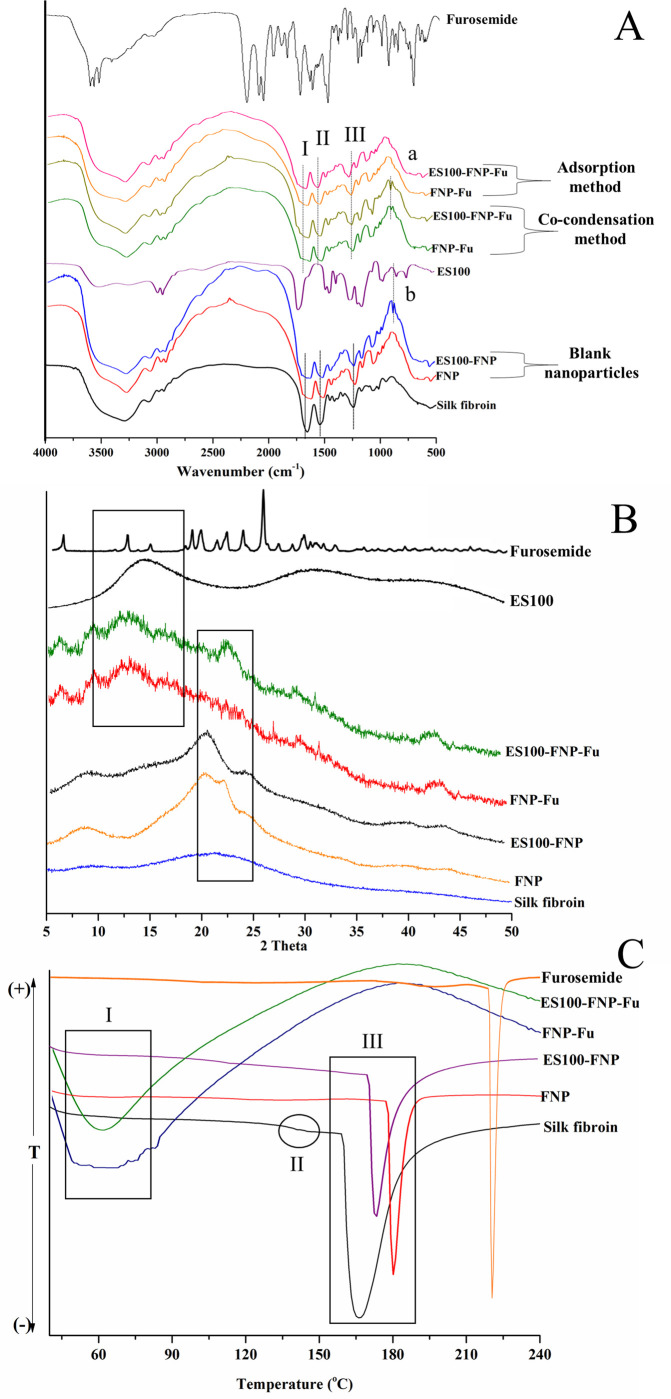
Analytical graphs of FNP-Fu, ES100-FNP-Fu, and other relevant compounds. (A) Fourier-transform infrared (FT-IR) spectra, the peak I, II, III, a, and b represents the fibroin amide I, amide II, amide III, furosemide aryl-chloro group, and ES100 C-H bending signal; (B) X-ray diffractograms; and (C) differential scanning calorimetric (DSC) graphs (C), the signal I, II, and III corresponds to the water evaporation, silk-I glass transition temperature, and material decomposition.

The particles structures and drug-material interactions between the fibroin, ES100, and furosemide were elucidated using FT-IR, XRD, and DSC techniques (**Fig 5**).

Firstly, the FT-IR spectra of FNP and ES100-FNP exhibited characteristic silk-II-fibroin peaks at ∼1626 cm^-1^ (C = O stretch), ∼1525 cm^-1^ (N-H bending), and ∼1240 cm^-1^ (C-N stretch), corresponding to the amide I, II, and III structures, respectively (**Fig 5A**). This demonstrated that both the formulation procedures were successful in converting the water-soluble silk-I fibroin structure in the solution to the water-insoluble silk-II structure in the particles [24]. Moreover, in the ES100-FNP spectra, the appearances of new peaks at ∼869 cm^-1^, corresponding to the ES100 C-H bending ∼1045 cm^-1^ (C-O stretching) [44, 46], confirmed the presence of ES100 in the NP structures. Additionally, the FNP-Fu and ES100-FNP-Fu spectra possessed the distinct furosemide peaks at 880 cm^-1^ (aryl-chloro group oscillation) [28], indicating the furosemide encapsulation. It is also worth to note that the fibroin amide-II and amide-III peaks slightly shifted to higher wavenumbers in furosemide loaded particles, compared to the blank ones. This fact suggests that the fibroin/ES100 has formed additional hydrogen bonding with furosemide at the nitrogen atom positions.

Secondly, the XRD diffractograms (**Fig 5B**) of the blank FNP and ES100-FNP exhibited silk-II structure by a sharp peak at 2θ of ∼21°, corresponding to a long-range crystallographic distance of the β-sheet of 4.5 Å [49–51]. The ES100 amorphous peak at 2θ of ∼13^o^ appeared in the ES100-FNP, indicating the success incorporation. Interestingly, the diffraction patterns of FNP-Fu and ES100-FNP-Fu showed two small broad peaks at ∼6.5^o^ and ∼23^o^, corresponding to those of the pure crystalline furosemide, confirming that the furosemide molecules were partly crystallized in the particles.

Thirdly, the DSC technique was used to evaluate the NP thermo-behaviors (**Fig 5C**). the peaks at ∼61.5°C showed the dehydration process of the samples, while the endothermic peaks of ∼170–180°C corresponded to the sample degradations [24]. Notably, the particles degradation temperature in our study was lower than that of other works, possibly due to the differences in the fibroin sources and structures [9]. The slight slope at ∼142°C was the glass transition temperature (T_g_) of the fibroin silk-I amorphous structure [24]. In the FNP and ES100-FNP formulas, the disappearance of this slope, together with the increases in the degradation temperature, re-confirmed the silk-II crystalline formation. Furthermore, the furosemide degradation peak of 210–220°C [52, 53] was absent in the DSC thermograms of FNP-Fu and ES100-FNP-Fu, indicating that the furosemide was covered by the NP materials, consequently enhances its thermo-stability.

In summary, the ES100 and furosemide were successfully incorporated into the FNP; the particles structures were mainly silk-II polymorph; the furosemide was partly crystallized in the NP that formed “durian thorns”, mentioned in previous sections; and the furosemide thermo-stability was enhanced.

### Hematotoxicity

The toxicity of FNP and ES100-FNP was respectively evaluated via hemolysis test on the sheep erythrocytes. The results indicated that all formulas showed no potential toxicity (i.e., cell lysing ability) to the erythrocytes at a concentration of as high as 10 mg/mL, with the %hemolysis of < 5%. Therefore, the particles were considered safe for the applications in human biological systems.

### *In vitro* drug release study

Recently, FNP have been utilized as a drug delivery system for oral administrations, which demonstrated potential abilities to overcome the pH variations in the gastrointestinal tract with limited drug release in the stomach and controllable drug release in the intestine [20, 28]. Hence, to widen the versatility of FNP in biomedical applications at different pH conditions, the ES100-FNP were developed. For this reason, the FNP-Fu and ES100-FNP-Fu, at two different formulation approaches of co-condensation and adsorption, were subjected to the *in vitro* release tests in (1) the gastrointestinal tract and (2) different pH of 6.8, 9.5, and 12. Then, the release kinetics were determined following standard models of zero-order, first-order, Higuchi, Korsmeyer-Peppas, and Hixson-Crowell. Finally, the particle structures, after the release tests, were investigated and compared with the initial structures, using the FT-IR spectroscopy.

**Fig 6:**
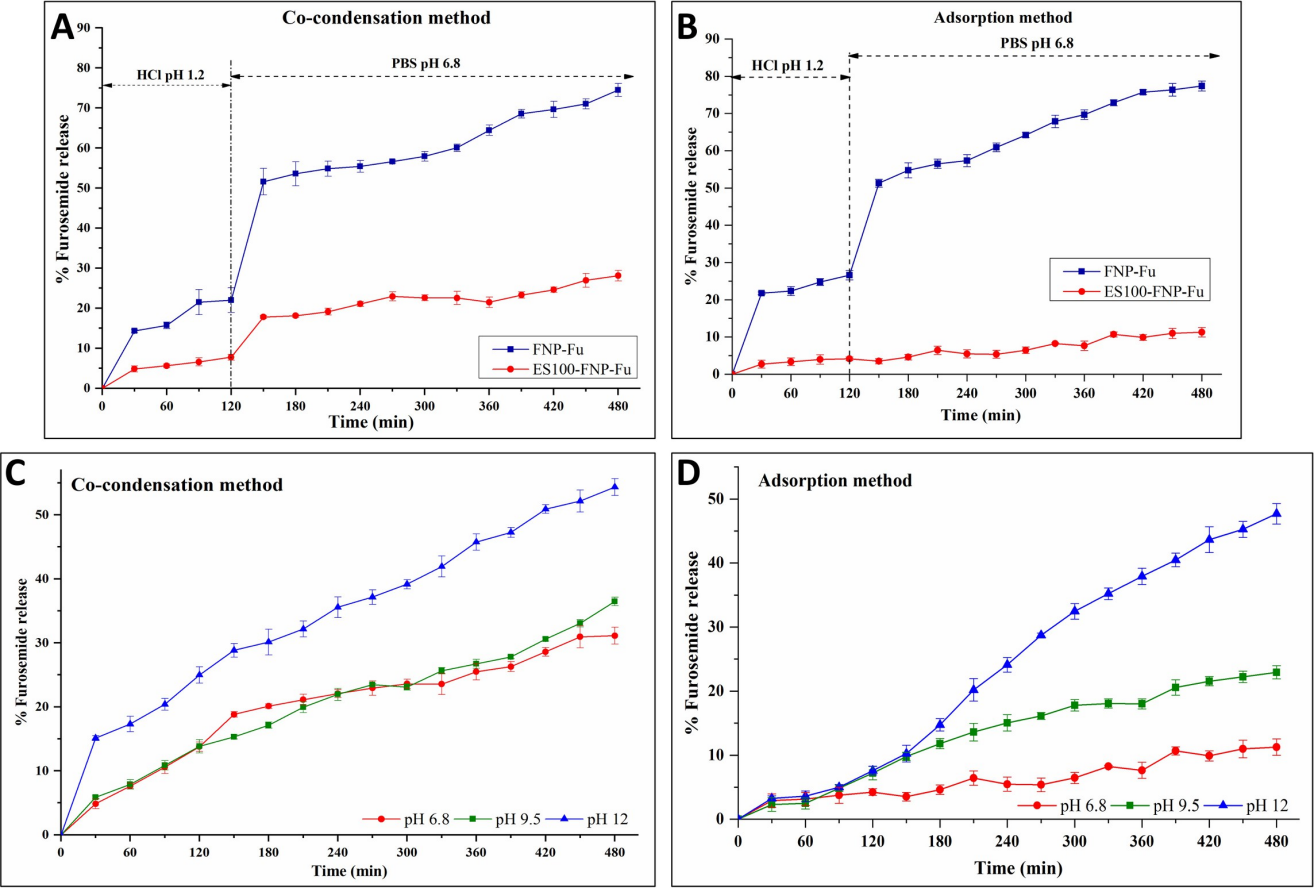
Furosemide release profiles in simulated gastrointestinal fluid at 37°C of FNP-Fu and ES100-FNP-Fu (A_,_ B), and in pH 6.8, 9.5, and 12, at 37°C, of ES100-FNP-Fu at two different encapsulation methods of co-condensation and adsorption (C, D).

Regarding the release profiles in the gastrointestinal tract (**Fig 6A and 6B**), all four formulas showed limited furosemide release within the first 2 h at pH 1.2 (i.e., stomach condition), followed by a burst release after subjecting to the intestine condition at pH 6.8, and a gradual release for the next 6 h, reaching the maximum accumulated furosemide release of ∼75% for FNP-Fu and ∼10–25% for ES100-FNP-Fu. ES100 is a pH-responsive polymer that highly dissolves at pH > 7, thus, at pH 1.2, ES100 was almost insoluble, providing a coating surface for the NP to minimize drug loss when passing through the stomach. Whereas, at pH 6.8, the ES100 was partly soluble in the media, thus, the particles structures were loosened and the drug was gradually released out from the NP. Conclusively, the ES100-FNP could be a potential delivery system for oral applications.

To further explore the ES100-FNP versatility, the particles were additionally subjected to different release media at pH 6.8, pH 9.5, and pH 12 (**Fig 6C and 6D**). Expectedly, at higher pH, the ES100 started to swell and dissolve, consequently letting more drug to be released out from the NP, following the order of pH 12 > pH 9.5 > pH 6.8. Moreover, as previously discussed, the pHpzc of the FNP and ES100-FNP was ∼5.0 and ∼5.4, respectively (**Fig 4**), therefore, at all three pH of 6.8, 9.5, and 12, the particles mainly possessed a negative charge. On the other hand, the furosemide had two pKa values of ∼3.2 and ∼10.2, thus, at pH 6.8 and 9.5, the drug had both negative (COO-) and positive (NH_3_^+^) charges, with the negative charge being dominant at higher pH. At pH 12, the drug was totally in anionic state. As such, the ionic interactions between the NP and furosemide become weaker as the pH increased, consequently making more drug released out from the particles at higher pH. This release characteristic allows for highly efficient drug release at the target with different pH (i.e., colon, cancer microenvironment, and inflammatory conditions).

Interestingly, the ES100-FNP-Fu different formulation processes significantly affected the furosemide release rates and kinetics. In all three investigated pH, particles formulated with the co-condensation method exhibited higher release rates than those with the adsorption method. This was because in the co-condensation process, furosemide was homogeneously distributed in the ES100-FNP in mainly molecular dispersion form, as discussed in previous sections. This fact, together with the particle smaller sizes and larger surface areas, leads to a faster drug release rate [54]. Moreover, the diffusion exponent (n) of the Korsmeyer-Peppas model of 0.45 < n < 0.9 indicated that the drug release followed an anomalous (non-Fickian) transport mechanism, by a combination of diffusion and dissolution (**Table 3**) [55]. In other words, the release of furosemide from ES100-FNP-Fu made by co-condensation method is controlled by the swelling, corrosion, and dissolution of ES100 in the medium. On the other hand, the ES100-FNP-Fu made by the adsorption method followed the zero-order kinetics, with slower and constant release rates [56]. This was due to the unique durian-shaped particles containing partially crystallized furosemide. In such setting, the amorphous furosemide was firstly dissolved by the medium molecules and “released out”, then, the crystallized furosemide was changing to the amorphous structure for equilibrium, followed by the release of these furosemide molecules. The process was repeated continuously, making the release rate stable and unaffected by the furosemide concentrations.

**Table 3:** Summary of R^2^ values of different kinetics models for the release of ES100-FNP-Fu at different pH of 6.8, 9.5, and 12, at two different encapsulation methods of co-condensation and adsorption.

Formulation method	R^2^ at different pH			Formulation method	R^2^ at different pH		
	6.8	9.5	12		6.8	9.5	12
**Co-condensation**				**Adsorption**			
Zero-order	0.9155	0.9734	0.9547	Zero-order	**0.9281**	**0.9676**	**0.9853**
First-order	0.9378	0.9813	0.9836	First-order	0.9271	**0.9767**	0.9824
Higuchi	0.9756	0.9722	**0.9888**	Higuchi	0.8761	0.9563	0.8909
Korsmeyer-Peppas	**0.9834**	**0.9915**	**0.9826**	Korsmeyer-Peppas	0.8710	0.8693	0.8483
Hixson-Crowell	0.9308	0.9797	0.9773	Hixson-Crowell	0.9273	0.9738	0.9846

**Fig 7:**
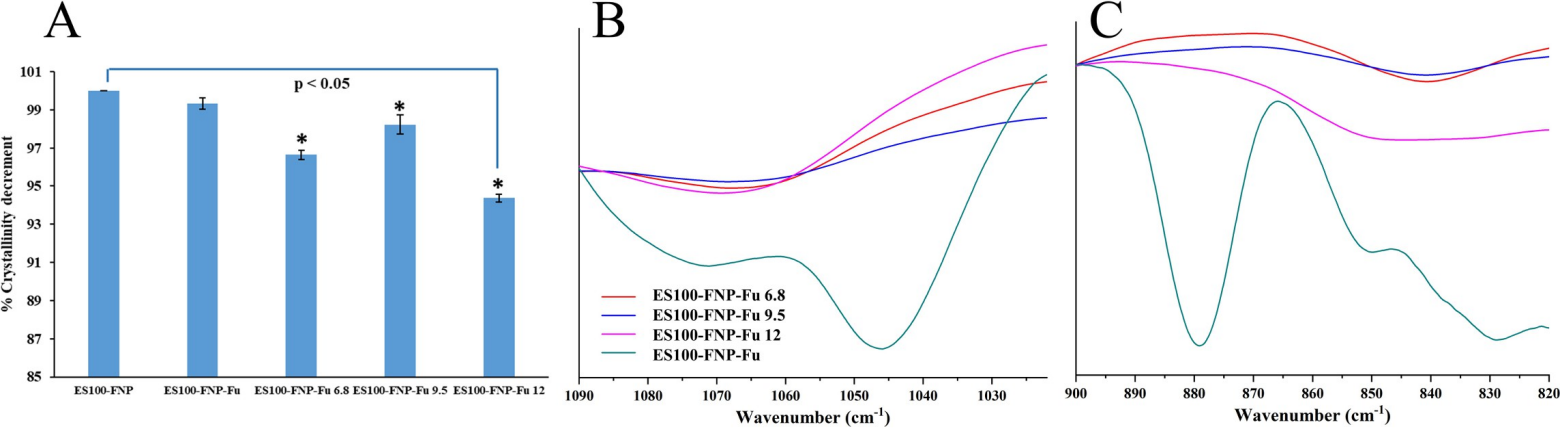
(A) Crystallinity decrement (%), in comparison with the blank ES100-FNP (100%), and Fourier-transform infrared (FT-IR) spectra, of the ES100-FNP-Fu before and after the release tests in different pH of 6.8, 9.5, and 12, at the peak of (B) ∼1045 cm^-1^ (ES100 C-O stretching), and (C) ∼880 cm^-1^ (furosemide aryl-chloro group oscillation).

Last but not least, after the release studies, the ES100-FNP-Fu had their structures significantly altered (**Fig 7**). The particles crystallinity decreased by ∼2–5%, suggesting that some parts of the particles were dissolved/disintegrated during the release tests (**Fig 7A**). This was further confirmed by the reduction in FT-IR intensities of the two characterized peaks of ES100 C-O stretching, at ∼1045 cm^-1^ (**Fig 7B**), and furosemide aryl-chloro group oscillation, at ∼880 cm^-1^ (**Fig 7C**). Therefore, it is concluded that during the release studies in different pH settings, the ES100 was swollen, corroded, and dissolved in the medium, consequently releasing the furosemide out from the particles.

In conclusions, the ES100-FNP could favorably control the release rates of the encapsulated drugs in different pH conditions, and these release rates could be potentially altered by adjusting the formulation methods. The zero-order drug release rate of the adsorption-particles is independent of the concentration of the drug in the NP, thus, a constant amount of drug is released per unit time, which is beneficial in case of requirement for steady drug levels in the bloodstream and a constant therapeutic effect. Whereas, the Korsmeyer-Peppas release model of the co-condensation-particles exhibits both burst release and sustained release characteristics, which could be employed for cases that need high initial drug level and maintained drug concentration afterward [43].

## Conclusions

In this study, the ES100-FNP was successfully synthesized as a novel pH-responsive drug delivery system, by two different methods of co-condensation and adsorption. Incorporating the model drug, zwitterionic furosemide (BCS class IV), the particles possessed nano-sizes of 200–350 nm, narrow polydispersity, and negative zeta potential of ∼ -20 mV, silk-II structures, enhanced thermo-stability, non-cytotoxic to the red blood cells, controllable drug entrapment efficiency of 30%-60%, and unique spherical/durian-like shapes, dependent on the formulation methods. Interestingly, under varying pH conditions of 6.8, 9.5, and 12, the ES100-FNP exhibited the ability to regulate the release rates of furosemide depending on the methods of formulation. For instance, when produced via the co-condensation method, the furosemide release profiles from ES100-FNP primarily relied on ES100 swelling and corrosion, adhering to the Korsmeyer-Peppas kinetics model. Conversely, ES100-FNP prepared through the adsorption method displayed consistent release rates following zero-order kinetics, attributed to the gradual dissolution of furosemide in the surrounding medium. In summary, the novel ES100-FNP demonstrated much potentials and versatilities as a pH-responsive drug delivery system in different pH environments.

## Supporting information

S1 Fig Molecular structures of (A) furosemide, (B) Eudragit S100 (ES100), and (C) silk fibroin (Gly: Glycine, Ser: Serine, Ala: Alanine).
(TIF)
