# Supplementary figures and images for: Development of pH-responsive Eudragit S100-functionalized silk fibroin nanoparticles as a prospective drug delivery system
Source: PLoS One. 2024 May 23;19(5):e0303177. doi: 10.1371/journal.pone.0303177 (PMC11115272; doi:10.1371/journal.pone.0303177)

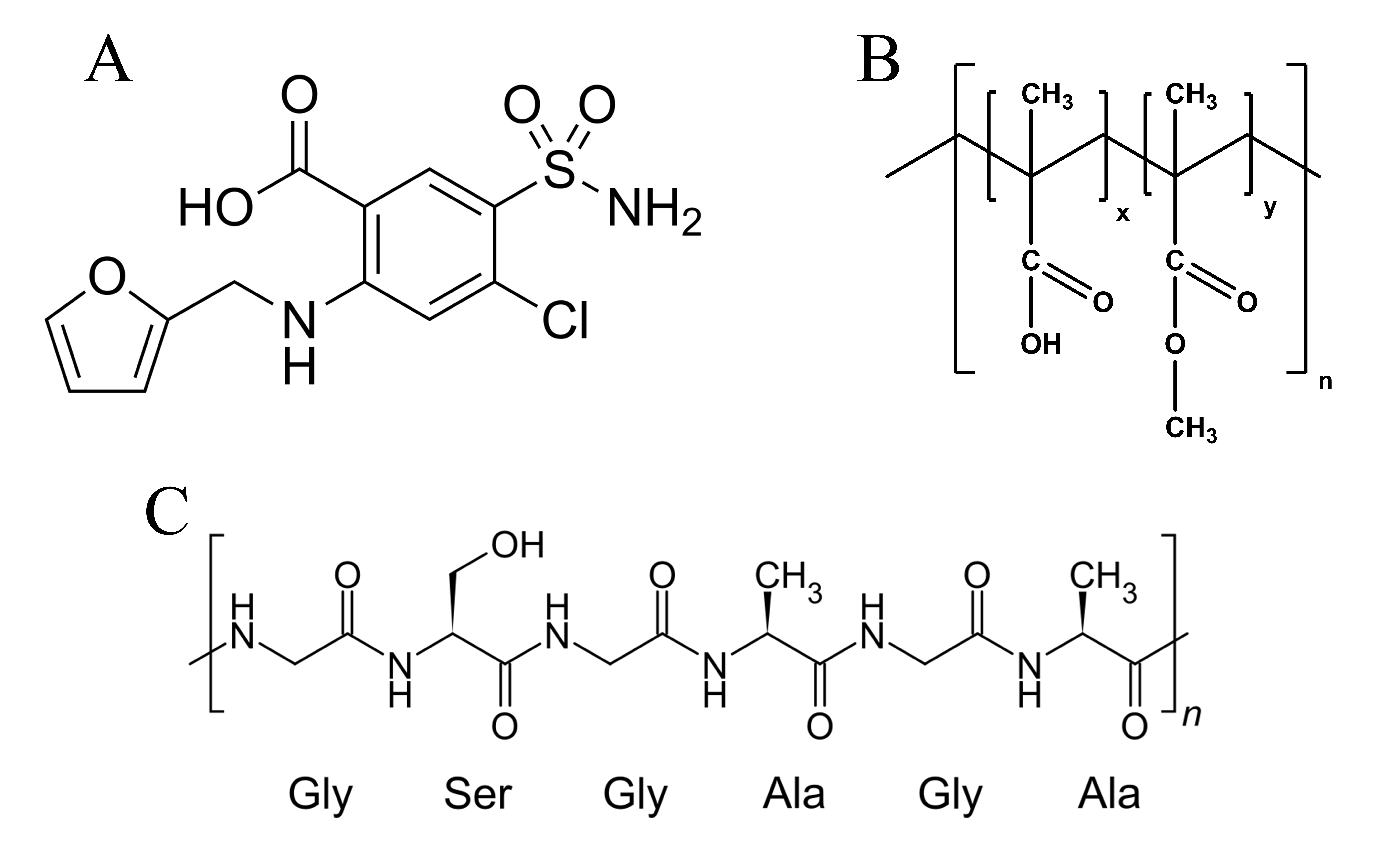

Supplement: S1 Fig — Molecular structures of (A) furosemide, (B) Eudragit S100 (ES100), and (C) silk fibroin (Gly: Glycine, Ser: Serine, Ala: Alanine). (TIF) [file pone.0303177.s001.tif]
